# P2X_7_ receptor activation may be involved in neuronal loss in the retinal ganglion cell layer after acute elevation of intraocular pressure in rats

**Published:** 2013-09-28

**Authors:** Tetsuya Sugiyama, So Yeon Lee, Taeko Horie, Hidehiro Oku, Shinji Takai, Hidetoshi Tanioka, Yumi Kuriki, Shota Kojima, Tsunehiko Ikeda

**Affiliations:** 1 Department of Ophthalmology, Osaka Medical College, Takatsuki, Osaka, Japan; 2 Nune Eye Hospital, Seoul, Korea; 3 Department of Pharmacology, Osaka Medical College, Osaka, Japan; 4 Nara Research and Development Center, Santen Pharmaceutical Co. Ltd., Takayama-cho, Ikoma-shi, Nara, Japan

## Abstract

**Purpose:**

To investigate whether the P2X_7_ receptor is involved in retinal ganglion cell (RGC) death after the intraocular pressure (IOP) is elevated in rats.

**Methods:**

After the IOP was elevated to 90 mmHg for 1 h, the rats were subsequently administered oxidized adenosine triphosphate (OxATP) and brilliant blue G (BBG) as P2X_7_ antagonists. The rats were euthanized 7 days after IOP elevation for histologic evaluation and at 1, 3, and 7 days after IOP elevation to immunostain for the P2X_7_ receptor and neuron-specific class III β-tubulin in the retina. Changes in P2X_7_ receptor expression were measured in total retina extracts using western blot analysis. Quantitative real-time PCR was also performed using the entire retina to determine whether the P2X_7_ receptor is involved in upregulating tumor necrosis factor (TNF)-α, interleukin (IL)-1β, and IL-6 at 1, 2, and 3 days after the IOP was elevated.

**Results:**

RGC density and the inner plexiform layer thickness significantly decreased 7 days after IOP elevation, but were dose-dependently preserved when treated with OxATP or BBG. P2X_7_ immunoreactivity in the RGCs increased after IOP elevation, with the peak occurring from day 1 through day 3. Protein levels of P2X_7_ receptor were significantly increased 1, 2, and 3 days after IOP elevation. The messenger ribonucleic acid expression of the P2X_7_ receptor, TNF-α, IL-1β, and IL-6 was significantly upregulated in the retina after IOP elevation, and was suppressed by treatment with OxATP.

**Conclusions:**

These results suggest the expression of the P2X_7_ receptor is upregulated in the retina after IOP elevation, leading to RGC death. Upregulation of TNF-α, IL-1β, and IL-6 might be involved in this mechanism of RGC death. Furthermore, P2X_7_ antagonists may prevent RGC death after IOP elevation.

## Introduction

P2X_7_ receptors were originally described in cells of hematopoietic origin (e.g., macrophages, microglia, and certain lymphocytes), and function in mediating the inﬂux of Ca^2+^ and Na^+^ ions and the release of proinﬂammatory cytokines. P2X_7_ receptors may affect neuronal cell death through their ability to regulate the processing and release of interleukin (IL)-1β, a key mediator in neurodegeneration and chronic inﬂammation [1-3]. Other studies have found that the activation of P2X_7_ receptors may be involved in the release of tumor necrosis factor (TNF)-α, IL-1β, and IL-6 from microglia and mast cells during mitosis, inflammation, and proliferation [4-7].

Several studies have demonstrated the expression of P2X_7_ receptors in retinal ganglion cells (RGCs) [8-10]. Other studies have reported that activation of P2X_7_ receptors might be involved in RGC death in vitro and in vivo through intracellular calcium increase [11-13]. However, the exact mechanisms for how activation of P2X_7_ receptors is related to RGC death remains unknown. Further, regarding cells other than RGCs, several studies have found an association between P2X_7_ receptors and TNF-α and several interleukins in apoptosis [14,15].

The focus of neuroprotective therapy in glaucoma has been preventing progressive RGC damage by intervening in neuronal death pathways. Several animal models, including those for acute and chronic intraocular pressure (IOP) elevation, optic nerve axotomy, and optic nerve crush, have been used for studies of neuroprotection in glaucoma [16].

In the present study, we aimed to determine whether the P2X_7_ receptor is involved in retinal neuronal loss, especially in the ganglion cell layer (GCL), after acute IOP elevation. First, we examined the effects of P2X_7_ antagonists—oxidized adenosine triphosphate (OxATP) [17] and brilliant blue G (BBG) [18]—on IOP elevation–induced histologic changes in the rat retina. Second, immunohistochemical studies regarding this receptor, TNF-α, and IL-1β were performed to verify their upregulation in the rat retina after IOP elevation. Third, real-time PCR was performed to investigate quantitatively the association of changes in the retinal messenger ribonucleic acid (mRNA) expression of this receptor and several cytokines after IOP elevation.

## Methods

### Animals and reagents

For this study, we used 10- to 12-week-old adult male Wistar rats (bodyweight, 200–260 g). The care of the animals and the experimental procedures conformed to the guidelines for the Care and Use of Laboratory Animals by the Institute for Laboratory Animal Research and the Association for Research in Vision and Ophthalmology (ARVO) Statement for the Use of Animals in Ophthalmic and Vision Research. Unless otherwise noted, the chemicals used in this study were purchased from Sigma-Aldrich (St. Louis, MO).

### Intraocular pressure elevation and drug administration

A 30 G infusion cannula was inserted into the anterior chamber of the left eye under systemic anesthesia with intraperitoneal pentobarbital (35 mg/kg bodyweight). This infusion cannula was connected to a bottle of phosphate-buffered saline (PBS; 0.9% sodium chloride, Otsuka, Tokyo, Japan) through a pressure transducer (P10EZ; Gould Statham Instruments, Hatorey, Puerto Rico) for continuous monitoring of actual IOPs. The IOP was artificially elevated to 90 mmHg for 60 min by increasing the height of the bottle. Red reflux from the fundus confirmed that complete retinal ischemia had not occurred at that IOP level. In a sham control eye, the IOP was maintained at 15 mmHg for 60 min. Immediately after the IOP elevation was completed, 5 µl of PBS or 5 µl of OxATP (1–100 µM) or BBG (0.3–300 nM) dissolved in PBS was injected intravitreally from the pars plana through a 30-gauge needle on a Hamilton syringe (701LT, Hamilton, Reno, NV).

### Histologic analysis

A total of 60 eyes from 60 rats were used for histologic analysis in this study. Eight eyes were used for the sham control, i.e., eight eyes were treated with PBS alone. Four, six, four, and six eyes were treated with OxATP at 3, 10, 30, and 100 µM, respectively; and four, seven, seven, and six eyes were treated with BBG at 0.3, 3, 30, and 300 nM, respectively. Seven days after IOP elevation, the rats were euthanized with inhalation of 100% carbon dioxide. The eyes were enucleated, fixed in Davidson’s solution overnight, rinsed with PBS, and embedded in paraffin. Transverse sections of the retina (3 µm thick) were cut with the optic nerve head (ONH) in the center, and stained with hematoxylin and eosin. For the analysis, four light photomicrographs (200X magnification) representing a 600 µm field were obtained approximately 1 mm from the center of the ONH in each retinal slice using a light microscope (ECLIPSE 80i, Nikon, Tokyo, Japan). A masked examiner (SL) then counted all GCL cells in the entire field, without excluding displaced amacrine cells, because it has been reported that the density of these cells does not affect the evaluation of RGC loss in experimental glaucoma [19]. The masked examiner also measured the thickness of the inner plexiform layer (IPL), the inner nuclear layer (INL), and the outer nuclear layer (ONL) at three different spots of each photomicrograph.

### Immunohistochemistry

For the immunohistochemical analyses in this study, three eyes from three rats were used for each condition: normal eyes and the eyes on days 1, 2, 3, and 7 after IOP elevation, as well as the eyes treated with OxATP at 30 µM just after IOP elevation. Rats were euthanized at 1, 2, 3, and 7 days after IOP elevation, as described. The eyes were enucleated, fixed in 4% paraformaldehyde in PBS overnight, rinsed with 30% sucrose, embedded in Tissue-Tek ornithine carbamoyltransferase (Sakura Finetechnical, Tokyo, Japan), and snap-frozen in liquid nitrogen. Then, 8-µm-thick frozen sections were cut with a cryostat (CM3000, Leica Biosystems, Wetzlar, Germany). For visualizing the expression of the P2X_7_ receptor in the retina, immunohistochemistry was performed using a rabbit primary antibody for the intracellular domain of the P2X_7_ receptor (APR-004, Alomone Labs, Jerusalem, Israel). For identifying the expression of the P2X_7_ receptor in neurons or microglia/macrophages, double immunostaining was performed for the P2X_7_ receptor and neuron-specific class III β-tubulin (TUJ1) or CD68. For this, mouse Alexa Fluor 488–labeled anti-TUJ1 monoclonal antibody (A488–435L, Covance Research Products, Princeton, NJ) and mouse anti-CD68 (MAB1435, Merck Millipore, Billerica, MA) were used. The sections were blocked in 5% normal goat serum and 2% bovine serum albumin (BSA) for 1 h, and then incubated with a rabbit anti-P2X_7_ receptor antibody (1:200) and mouse anti-TUJ1 (1:100) or anti-CD68 (1:50) antibody at 4 °C overnight. Then, the samples were incubated for 2 h at room temperature with fluorescently labeled secondary antibodies (1:500; Alexa Fluor 594, antirabbit; Alexa Fluor 488, antimouse only for anti-CD68; Life Technologies, Carlsbad, CA). In addition, for visualizing the expression of TNF-α or IL-1β in microglia/macrophages in the retina, double immunostaining of CD68 with TNF-α or IL-1β was performed. After rats were perfused through the heart with saline under deep anesthesia followed by 4% paraformaldehyde in PBS (pH 7.4), the eyes were enucleated, fixed, and embedded, followed by cutting into frozen sections as described above. Primary antibodies for TNF-α (goat polyclonal, sc-1351, Santa Cruz Biochemistry, Santa Cruz, CA) and IL-1β (rabbit polyclonal, ab9787, Abcam, Cambridge, UK) were used. After blocking with 2% BSA for 1 h, the sections were incubated overnight with a mouse anti-CD68 antibody (1:100) as well as a goat anti-TNF-α (1:50) or rabbit anti-IL-1β (1:500) antibody at 4 °C. Next, the samples were incubated for 2 h at room temperature in secondary antibodies (1:500; Alexa Fluor 594, antigoat or antirabbit; Alexa Fluor 488, antimouse; Life Technologies). As a negative control, the sections were incubated only with solvent instead of diluted solution of primary antibodies. The nuclei were stained with 4’,6-diamino-2-phenylindole dihydrochloride (1:500), and the images were acquired using a fluorescence microscope (BX50, Olympus, Tokyo, Japan).

### Western blot analysis for P2X_7_ receptors

Three eyes of three rats were used for each condition: normal eyes, the eyes on days 1, 2, and 3 after IOP elevation as well as the sham control eyes on day 1. The entire retina was isolated from each eye after the rat was euthanized, and homogenized with a mechanical homogenizer in five pellet volumes of lysis buffer containing 50 mM Tris-hydrochloride (pH 7.6), 150 mM NaCl, 20 mM EDTA, 0.1% sodium dodecyl sulfate, 1.0% Nonidet P40, 1.0% sodium deoxycholate, 1.0 mM phensxylmethanesulfonulfluoride, 10 μM aprotinin, 10 μM leupeptin, and 10 μM pepstatin A. The suspension was centrifuged, and the supernatant was used to determine the protein concentration with a DC protein assay reagent (Bio-Rad, Hercules, CA). Samples containing 5 μg of protein were run on a 10% sodium dodecyl sulfate–polyacrylamide gel electrophoresis gel and electroblotted onto a polyvinyldene difluoride membrane (Bio-Rad). Blots were blocked for 1 h with 5% skim milk in Tris buffered saline containing 0.1% Tween-20 (TBS-T, pH 7.4), incubated for 2 days at 4 °C with a rabbit anti-P2X_7_ receptor antibody (1:500; APR-004, APR-008, Alomone Labs) or anti-α-tubulin antibody (1:1,000; Merck Millipore, Billerica, MA), and washed with TBS-T. Then blocking with 5% skim milk in TBS-T was done for 1 h, followed by incubation with the peroxidase-conjugated secondary antibodies (1:2,000; antimouse or antirabbit immunoglobulin G, Promega, Madison, WI) for 2 h at room temperature and washing with TBS-T. Immunoblots were developed with an enhanced chemiluminescence plus western blotting detection system (GE Healthcare, Little Chalfont, England). The densities of the bands of proteins were quantified with a luminescent image analyzer (LAS-3000, Fujifilm, Tokyo, Japan). The amount of protein expression was quantified using the equipped software (Multi Gauge version 3.0), and the blots were then reprobed with α-tubulin to confirm equal loading of protein into each well.

### Ribonucleic acid extraction and reverse transcription

For RNA extraction in this study, 28 eyes of 28 rats were used. Four eyes were used for each condition (days 1 and 3 after IOP elevation or the sham treatment, and day 2 after IOP elevation and the subsequent injection with OxATP at 1, 10, and 100 µM), with the exception of five eyes used only for day 2 after IOP elevation. At 1, 2, and 3 days after IOP elevation, the rats were euthanized as described above. The entire retina was removed from each eye, and total RNA was isolated from each retina using the PureLink RNA Mini kit (Life Technologies), according to the manufacturer’s protocol. Zirconia balls (YTZ-5, As-one, Osaka, Japan) and Tissue Lyser (Qiagen, Valencia, CA) were used to homogenize the retina, and the total RNA quality and quantity were assessed using a BioSpectrometer (Eppendorf, Hamburg, Germany). For critical RNA purification, DNase I (amplification grade; Life Technologies) was used. RNA was reverse-transcribed into cDNA using Thermal Cycler PCR System 9700 (Applied Biosystems, Foster City, CA) and SuperScript VILO Master Mix (Life Technologies), according to the manufacturer’s protocol, except that 16 μl of the RNA sample was used for 4 μl of the Master Mix.

### Quantitative real-time polymerase chain reaction

Quantitative real-time PCR (qPCR) analysis was performed using StepOne Plus System, TaqMan Fast Advanced Master Mix, and Assay-by-Design primers and probes (Applied Biosystems), according to the manufacturer’s instructions. The primer/probes used were as follows: P2X_7_ receptor Rn00570451_m1, TNF-α Rn01525859_g1, IL-1β Rn00580432_m1, and IL-6 Rn01410330_m1. For determining the cycle threshold (Ct) values, the threshold level of the fluorescence was set manually during the early phase of PCR amplification. The relative quantities of mRNAs were calculated using the 2^−ΔΔCt^ analysis method [20], with β-actin (Rn00667869_m1) as the endogenous control.

### Data analysis and statistical evaluation

Data are represented as the means±standard deviation (SD) or standard error of the mean (SEM). Statistical analysis was performed with an unpaired Student *t* test or Mann–Whitney U-test.

## Results

### Histologic analysis of effects of a P2X_7_ antagonist on intraocular pressure elevation–induced changes

**Figure 1:**
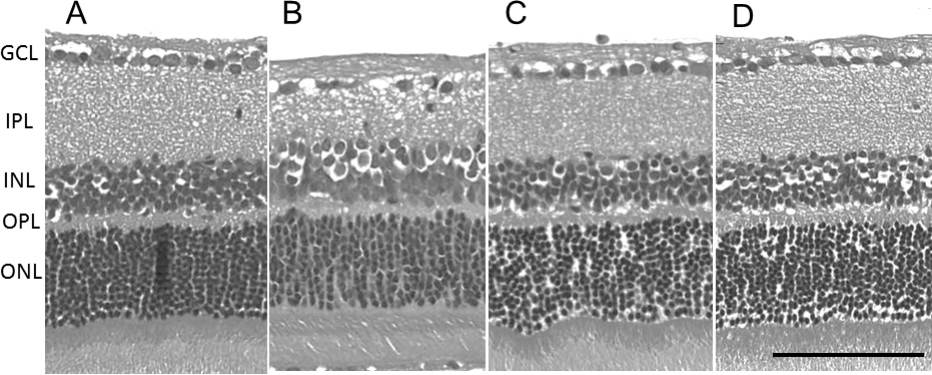
Photomicrographs of transverse sections of the posterior retina stained with hematoxylin and eosin. Sections were obtained from a sham control eye (**A**) and from eyes after intraocular pressure (IOP) elevation and intravitreal injection of phosphate-buffered saline (PBS; **B**) or the P2X_7_ antagonists—oxidized adenosine triphosphate (OxATP; 30 µM; **C**) and brilliant blue G (BBG; 30 nM; **D**). Cell loss in the ganglion cell layer (GCL) and thinning of the inner plexiform layer (IPL) were observed in the eyes after IOP elevation and PBS, but were rescued by P2X_7_ antagonists. Bar=100 µm.

**Figure 2:**
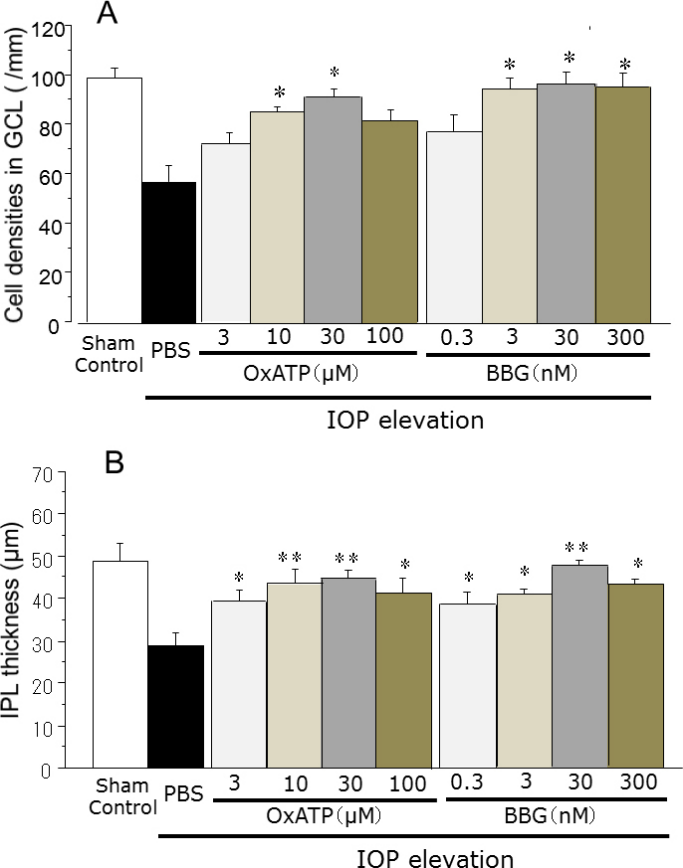
Effects of the P2X_7_ antagonists—oxidized adenosine triphosphate (3–100 µM) and brilliant blue G (0.3–300 nM)—on the retinal changes after IOP elevation. **A**: Cell density in the ganglion cell layer (GCL), **B**: inner plexiform layer (IPL) thickness. Data are expressed as the mean±standard error of the mean (SEM; n=4–8). The cell density in the eyes injected with phosphate-buffered saline (PBS) after intraocular (IOP) elevation was significantly lower than that in the sham control eyes (unpaired Student *t* test, p<0.01 for **A**, p<0.05 for **B**). The asterisks indicate significant differences in eyes after IOP elevation and intravitreal injection of PBS (unpaired Student *t* test, **p<0.01, *p<0.05).

Cell loss in the GCL and thinning of the IPL were observed in the eye treated with IOP elevation and PBS, compared with the sham control eye. However, these effects seemed to be ameliorated by intravitreal injections of the P2X_7_ antagonists—OxATP and BBG (Figure 1). The GCL cell densities and the IPL thickness were significantly lower in the eyes injected with PBS after IOP elevation than in the sham control eyes. Treatment with the P2X_7_ antagonists significantly increased GCL cell survival and preserved the IPL thickness (p<0.05, Figure 2). No significant decrease in the thickness of the INL or the ONL after IOP elevation was observed (p>0.05, data not shown).

### Immunohistochemical examination of P2X_7_ receptors after intraocular pressure elevation

**Figure 3:**
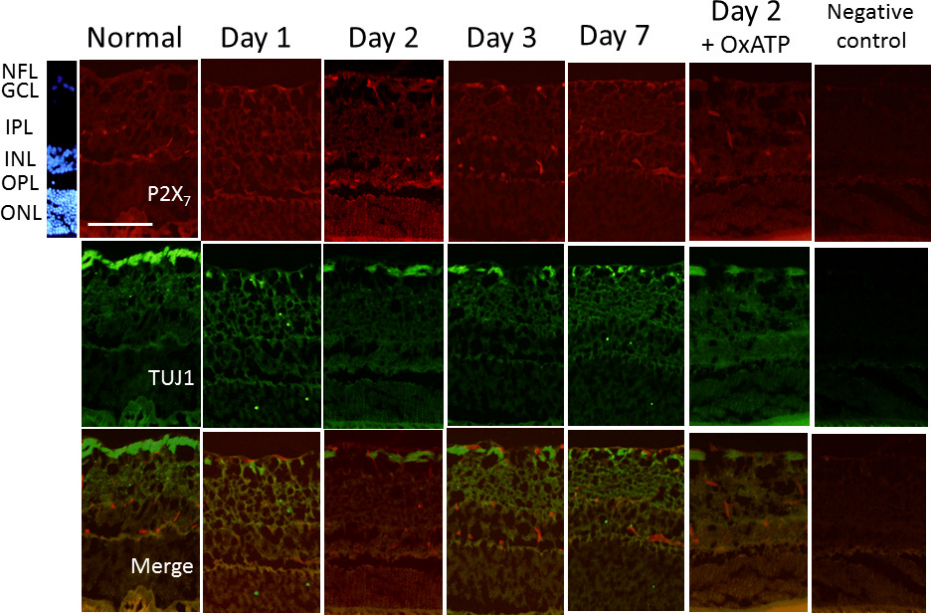
Representative photomicrographs of double immunostaining of the P2X_7_ receptor and neuron-specific class III β-tubulin (TUJ1) in the normal retina and in the retina on days 1, 2, 3, and 7 after intraocular pressure (IOP) elevation. Immunoreactivity of the P2X_7_ receptor was upregulated after IOP elevation, and the ganglion cell layer (GCL) cells were costained with anti-P2X_7_ receptors and anti-TUJ1, especially in the retina on day 2. Upregulation of the P2X_7_ receptor was suppressed by treatment with oxidized adenosine triphosphate (OxATP; 30 µM). Bar=100 µm.

**Figure 4:**
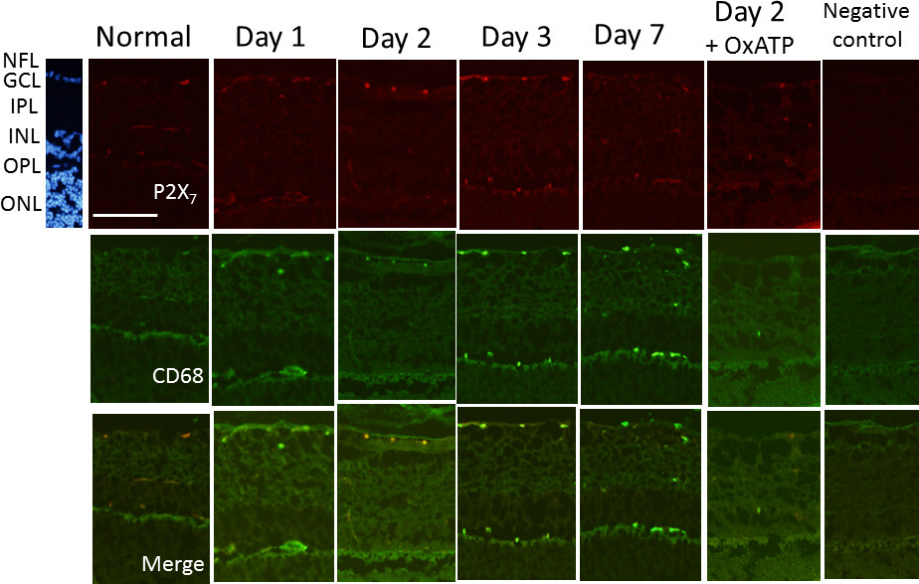
Representative photomicrographs of double immunostaining of the P2X_7_ receptor and CD68 in the normal retina and in the retina on days 1, 2, 3, and 7 after intraocular pressure elevation. P2X_7_-positive cells were also stained with anti-CD68 in the ganglion cell layer (GCL). Bar=100 µm.

**Figure 5:**
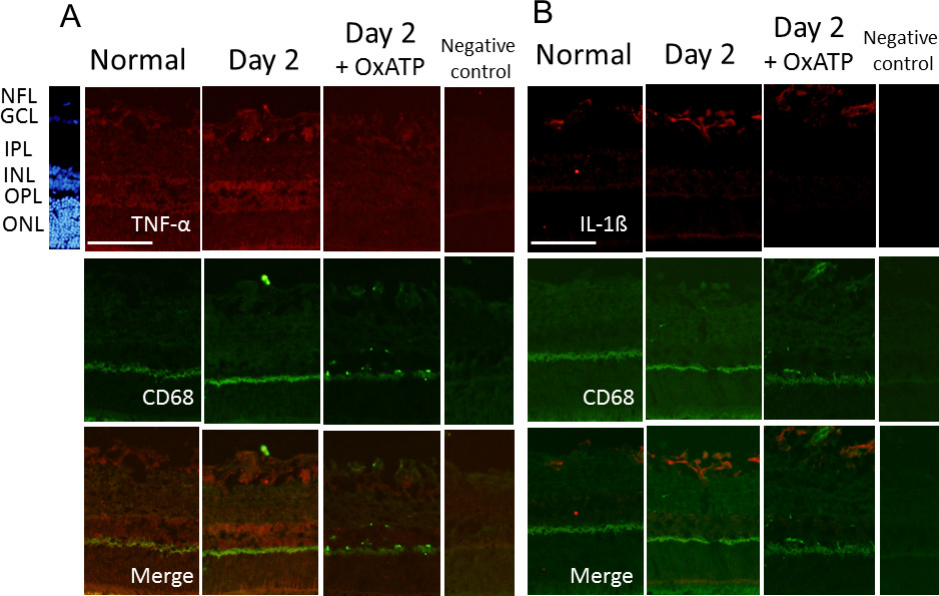
Representative photomicrographs of double immunostaining of CD68 and TNF-α or interleukin-1β (IL-1 β) in the normal retina and the retina on day 2, with and without treatment of oxidized adenosine triphosphate (OxATP) at 30 µM just after intraocular pressure (IOP) elevation. A: TNF- α, B: IL-1 β. Immunoreactivities of tumor necrosis factor-α (TNF-α) and IL-1β were upregulated in the ganglion cell layer (GCL) and inner plexiform layer (IPL) cells on day 2 after IOP elevation; they were subsequently suppressed by treatment with OxATP. Bar=100 µm.

On days 1, 2, 3, and 7, the expression of the P2X_7_ receptor was upregulated in cells of the GCL, IPL, and INL, compared with the normal retina. Double staining of TUJ1 and the P2X_7_ receptor showed that the expression of P2X_7_ was also upregulated in the TUJ1-positive cells in the GCL on days 1, 2, and 3 after IOP elevation (Figure 3). Upregulated expression of the P2X_7_ receptor was suppressed by treatment with OxATP (Figure 3). On days 1, 2, and 3, upregulated immunoreactivity of the P2X_7_ receptor was observed in CD68-positive cells, especially in the GCL (Figure 4). The immunoreactivities of TNF-α and IL-1β were upregulated in the CD68-positive cells of the GCL and IPL on day 2 after IOP elevation, which were then suppressed by treatment with OxATP (Figure 5).

### Protein levels of P2X_7_ receptor after intraocular pressure elevation

**Figure 6:**
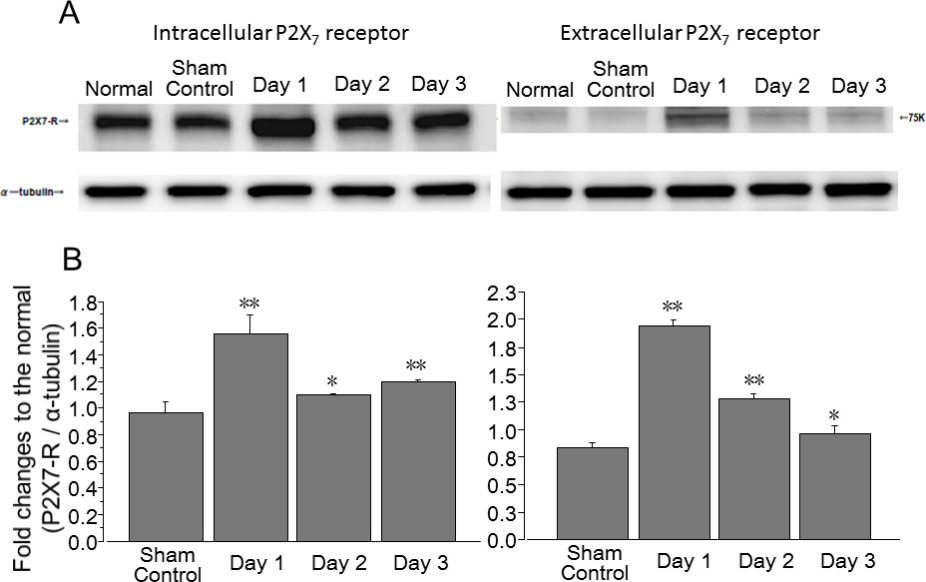
Western blot analysis of P2X_7_ receptor protein levels in the whole retina after IOP elevation. **A**: Representative immunoreactive bands of intracellular and extracellular P2X_7_ receptor in the normal, the sham control, and the retina on days 1, 2, and 3 after intraocular pressure (IOP) elevation. **B**: Densitometric quantification of immunoreactive bands of intracellular and extracellular P2X_7_ receptor in the sham control and the retina on days 1, 2, and 3 in comparison with normal retina. Data are expressed as mean±standard deviation (SD; n=3). The asterisks indicate significant differences from the sham control eyes (unpaired Student *t* test, **p<0.01, *p<0.05).

The protein levels of the intracellular and extracellular P2X_7_ receptor were significantly increased on days 1, 2, and 3 after IOP elevation compared to the sham control (p<0.01, p<0.05, p<0.01 and p<0.01, p<0.01, p<0.05, respectively; Figure 6).

### Messenger ribonucleic acid levels of P2X_7_ receptor, tumor necrosis factor-α and interleukins after intraocular pressure elevation

**Figure 7:**
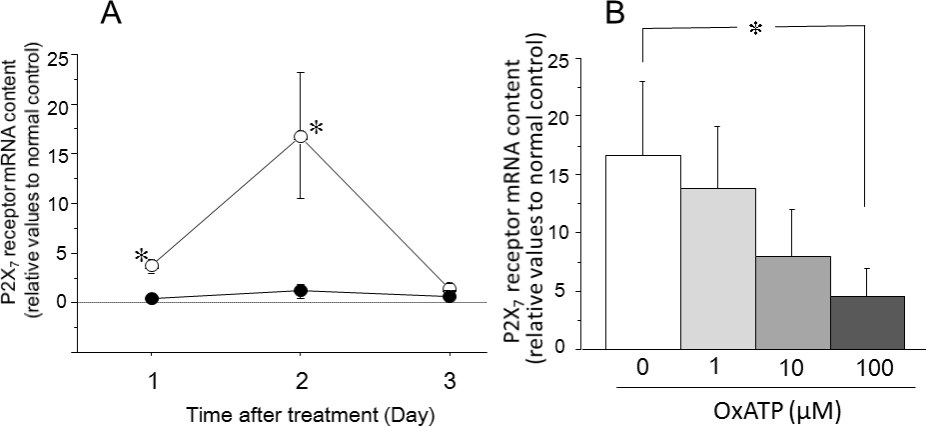
Effects of intraocular pressure (IOP) elevation on the P2X_7_ receptor messenger RNA (mRNA) content in the retina. **A**: Alterations in P2X_7_ receptor mRNA content (relative values to the normal control) in the retina after IOP elevation (open circles) or the retina after the sham treatment (closed circles). **B**: Effects of oxidized adenosine triphosphate (OxATP) on P2X_7_ receptor mRNA content in the retina 2 days after IOP elevation. Data are expressed as mean± standard error of the mean (SEM; n=4–5). The asterisks indicate significant differences from the sham control eyes (**A**) or from the eyes without OxATP treatment (**B**; Mann–Whitney U-test, *p<0.05).

**Figure 8:**
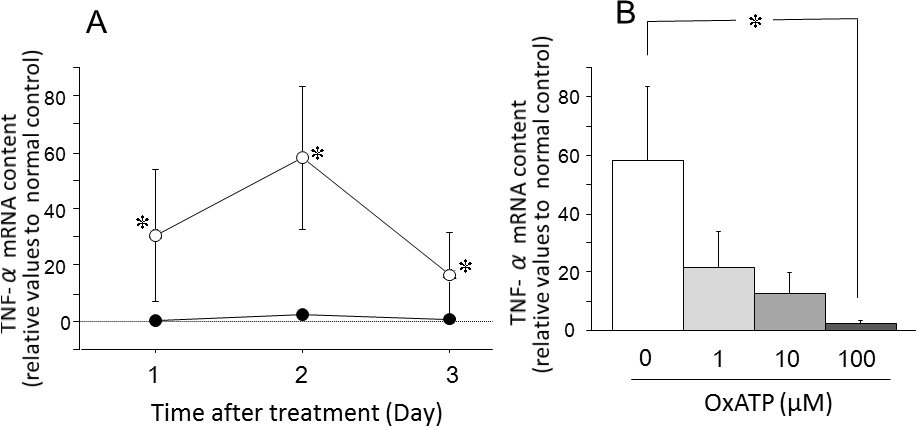
Effects of intraocular pressure (IOP) elevation on the tumor necrosis factor-α (TNF-α) messenger ribonucleic acid (mRNA) content in the retina. **A**: Alterations in TNF-α mRNA levels in the retina after IOP elevation (open circles) or the retina after the sham treatment (closed circles). **B**: Effects of oxidized adenosine triphosphate (OxATP) on TNF-α mRNA content in the retina 2 days after IOP elevation. Data are expressed as mean±standard error of the mean (SEM; n=4–5). The asterisks indicate significant differences from the sham control eyes (**A**) or from the eyes without OxATP treatment (**B**; Mann–Whitney U-test, *p<0.05).

**Figure 9:**
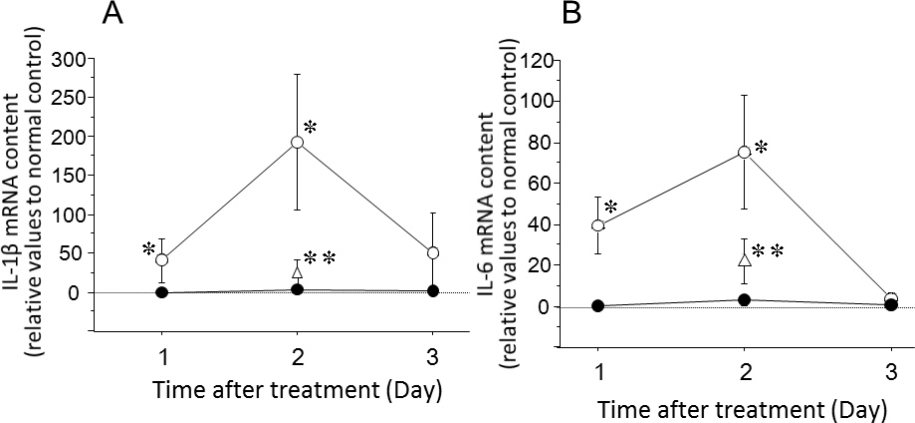
Effects of intraocular pressure (IOP) elevation on the messenger RNA (mRNA) content of interleukin-1β (IL-1β) and IL-6 in the retina. **A**: Alterations in IL-1β mRNA content in the retina after IOP elevation (open circles) or the retina after the sham treatment (closed circles). **B**: Alterations in IL-6 mRNA content in the retina after IOP elevation (open circles) or the sham treatment (closed circles). Open triangles represent samples from eyes treated with oxidized adenosine triphosphate (OxATP) at 10 µM just after IOP elevation. Data are expressed as mean±standard error of the mean (SEM; n=4–5). The asterisks indicate significant differences from the sham control eyes (*) or from the eyes after IOP elevation (**; Mann–Whitney U-test, p<0.05).

The expression of P2X_7_ receptor mRNA was significantly increased on days 1 and 2 after IOP elevation—treatment with a P2X_7_ antagonist decreased its expression in a dose-dependent manner (p<0.05, Figure 7). The expression of TNF-α mRNA was significantly increased on days 1–3 after IOP elevation. This increase was also significantly suppressed by the P2X_7_ antagonist (p<0.05, Figure 8). The expression of IL-1β and IL-6 mRNA was significantly enhanced on days 1 and 2 after IOP elevation. Those enhancements were also inhibited by the P2X_7_ antagonist (p<0.05, Figure 9).

## Discussion

The results of the current study revealed that the expression of the P2X_7_ receptor is upregulated in the retina after IOP elevation and that P2X_7_ antagonists ameliorated the IOP elevation–induced neuronal loss in the GCL. Our results also suggest that upregulation of TNF-α, IL-1β, and IL-6 might be involved in the mechanism underlying P2X_7_ receptor-mediated damage.

In the present study, we used an artificial IOP elevation rat model, since the damage is primarily induced in the inner retina in rats, similar to the scenario observed in human glaucoma [21]. In a previous ischemia-reperfusion injury model, the IOP level was set at over 110 mmHg [22-24]. However, in this study, we set the IOP level at 90 mmHg because the blood supply to the retina does not completely stop at this level, thus preventing the changes from being too dramatic, and, consequently, different from those seen in human chronic glaucoma. Indeed, no significant change in the INL thickness was detected, although the IPL thickness and cell density in the GCL were significantly decreased in our model. The results of the current study showed that deficient blood supply in an acute IOP elevation model also induced significant changes in the histology and inflammatory factors, including TNF-α, IL-1β, and IL-6, in the retina. In addition, 57% reduction was seen in GCL cell density at 7 days after IOP elevation in the present study. We recently reported that RGC density was decreased to 61% at 7 days after optic nerve crush injury [25]. Though we cannot simply compare these studies since the counting methods differ, similar rates of neuronal loss were seen in these different models.

In this study, immunohistochemical and quantitative real-time PCR analyses revealed the upregulation of the P2X_7_ receptor in neurons as well as microglia/macrophages in the GCL of a rat model of acute IOP elevation. To the best of our knowledge, this has not been previously reported. Several studies have reported the involvement of this receptor in regulating aqueous humor outflow [26] and the death of RGCs and retinal neurons [27-30]. Moreover, another study found that patients who had acute IOP elevation due to primary angle-closure glaucoma had remarkably increased ATP levels in the aqueous humor [31]. In addition, a study using bovine retinal eyecups revealed that a step increase of 20 mmHg in the hydrostatic pressure induced a threefold increase in vitreal ATP concentrations and that the ATP levels correlated with the degree of pressure increase [32]. Taken together, excess extracellular ATP may be linked to the activation of the P2X_7_ receptor after acute IOP elevation.

Our results also indicate that IOP elevation resulted in increased levels of retinal TNF-α, IL-1β, and IL-6, especially in microglia/macrophages in the GCL, which peaked 2 days after IOP elevation. Ischemia has been reported to result in increased levels of retinal TNF-α, which may play a deleterious role in retinal injury [33,34]. Studies using human glaucomatous eyes found that TNF-α and its receptor were upregulated in the glaucomatous ONH [35,36]. One of these studies also indicated that TNF-α might contribute to the progression of optic nerve degeneration through a direct effect on the axon of the RGCs by inducing nitric oxide synthase-2 in astrocytes [35]. Others have reported that transient retinal ischemia dramatically induced upregulation of IL-1β, which may mediate retinal injury [37,38]. Furthermore, IL-6 has been reported to be upregulated after retinal ischemia and to protect RGCs from ischemic injury [39,40]. In contrast, however, early IOP-induced injury in the optic nerve head is characterized by increased IL-6 expression [41].

Our current results suggest that the P2X_7_ antagonists—OxATP and BBG—may play an important role in preventing cell death induced by IOP elevation in various ocular diseases, including glaucoma. Based on the present study, it is not clear whether P2X_7_ antagonists exert neuroprotective effects directly on RGC, indirectly through inhibition of microglia/macrophage’s release of inflammatory mediators, or both. Inflammatory cytokines, including TNF-α and IL-1β, have been shown to contribute directly to the retinal injury [35,38]. We previously reported the protective effects of P2X_7_ antagonists in optic nerve crush injury [25] although the extent of the protection was rather weak compared to the present study. In the same study, we also revealed that P2X_7_ antagonists rescued RGCs in culture. Recently, P2X_7_ receptor activation was shown to mediate RGC death in a human retina model of ischemic neurodegeneration [42]. Another study also reported the potential neuroprotective effect of BBG on photoreceptor cell death using primary retinal cell cultures [43]. In addition, OxATP has been reported to inhibit apoptosis in cultured human retinal pigment epithelium [44]. Taken together, the neuroprotective effects of P2X_7_ antagonists might be direct and indirect.

Many mechanisms might be responsible for RGC injury induced by elevated IOP. The excessive pressure can damage the RGC soma directly, but it can also initiate damage by compressing RGC axons, which may interfere with intra-axonal transport of prosurvival molecules, such as trophic factors. Alternatively, pressure-induced compression of the retinal blood vessels can cause mild ischemia in retinal tissues. One limitation of our study is that we did not clarify the exact mechanisms underlying IOP elevation–induced damage involving the P2X_7_ receptor, although we showed the involvement of inflammatory responses by TNFα, IL-1β, and IL-6, which were probably induced by upregulation of this receptor [45,46]. It was also shown that inhibiting P2X_7_ receptor-activated NACHT, LLR, and PYD domains-containing protein 3 inflammasome formation and the consequent IL-1β release from glia preserve neuronal viability [47]. Another study has suggested that phosphorylation of p38 mitogen-activated protein kinase might play a role in axotomy-induced apoptosis of RGCs [48]. Further studies must be performed to delineate the detailed molecular pathway during IOP elevation–induced damage.

Another limitation is that other methods of P2X_7_ antagonist application have not yet been tested. For clinical applications, the effects of other methods, including instillation, should be investigated in the future. In addition, the safety of the P2X_7_ antagonist OxATP for human eyes should be evaluated. BBG is a dye used clinically to stain the internal limiting membrane in vitreoretinal surgeries, and its toxicity to a human Müller cell line has already been examined. Kawahara et al. [49] observed that apoptosis was not induced after exposure to BBG at 0.25 mg/ml (0.3 mM), which is much higher than the concentration used in the current study. It will be interesting to evaluate the safety of OxATP in the future.

In conclusion, the current study suggests that the expression of the P2X_7_ receptor in the retina, especially in neurons and microglia/macrophages in the GCL, is upregulated after IOP elevation and that P2X_7_ antagonists may prevent neuronal loss in the GCL. In addition, TNF-α, IL-1β, and IL-6 might be involved in this mechanism.

## References

[1] Skaper SD, Debetto P, Giusti P (2010). The P2X_7_ purinergic receptor: from physiology to neurological disorders.. FASEB J.

[2] Ferrari D, Chiozzi P, Falzoni S, Dal Susino M, Melchiorri L, Baricordi OR, Di Virgilio F (1997). Extracellular ATP triggers IL-1 beta release by activating the purinergic P2Z receptor of human macrophages.. J Immunol.

[3] Ferrari D, Chiozzi P, Falzoni S, Hanau S, Di Virgilio F (1997). Purinergic modulation of interleukin-1β release from microglial cells stimulated with bacterial endotoxin.. J Exp Med.

[4] Morigiwa K, Quan M, Murakami M, Yamashita M, Fukuda Y (2000). P2 Purinoceptor expression and functional changes of hypoxia-activated cultured rat retinal microglia.. Neurosci Lett.

[5] Friedle SA, Brautigam VM, Nikodemova M, Wright ML, Watters JJ (2011). The P2X_7_-Egr pathway regulates nucleotide-dependent inflammatory gene expression in microglia.. Glia.

[6] Zou J, Vetreno RP, Crews FT (2012). ATP-P2X_7_ receptor signaling controls basal and TNFα-stimulated glial cell proliferation.. Glia.

[7] Bernardino L, Balosso S, Ravizza T, Marchi N, Ku G, Randle JC, Malva JO, Vezzani A (2008). Inflammatory events in the hippocampal slice cultures prime neuronal susceptibility to excitotoxic injury: a crucial role of P2X_7_ receptor-mediator.. J Neurochem.

[8] Brändle U, Kohler K, Wheeler-Schilling TH (1998). Expression of the P2X_7_-receptor subunit in neurons of the rat retina.. Brain Res Mol Brain Res.

[9] Wheeler-Schilling TH, Marquordt K, Kohler K, Guenther E (2001). Jabs. Identification of purinergic receptors in retinal ganglion cells.. Brain Res Mol Brain Res.

[10] Ishii K, Kaneda M, Li H, Rockland KS, Hashikawa T (2003). Neuron-specific distribution of P2X_7_ purinergic receptors in the monkey retina.. J Comp Neurol.

[11] Zhang X, Zhang M, Laties AM, Mitchell CH (2005). Stimulation of P2X_7_ receptors elevates Ca^2+^ and kills retinal ganglion cells.. Invest Ophthalmol Vis Sci.

[12] Hu H, Lu W, Zhang M, Zhang X, Argall AJ, Patel S, Lee GE, Kim YC, Jacobson KA, Laties AM, Mitchell CH (2010). Stimulation of the P2X_7_ receptor kills rat retinal ganglion cells in vivo.. Exp Eye Res.

[13] Zhang X, Zhang M, Laties AM, Mitchell CH (2006). Balance of purines may determine life or death of retinal ganglion cells as A3 adenosine receptors prevent loss following P2X_7_ receptor stimulation.. J Neurochem.

[14] Wang Q, Wang L, Feng Y-H, Li X, Zeng R, Gorodeski GI (2004). P2X_7_ receptor-mediated apoptosis of human cervical epithelial cells.. Am J Physiol Cell Physiol.

[15] Schneider EM, Vorlaender K, Ma X, Du W, Weiss M (2006). Role of ATP in trauma-associated cytokine release and apoptosis by P2X_7_ ion channel stimulation.. Ann N Y Acad Sci.

[16] Danesh-Meyer HV (2011). Neuroprotection in glaucoma: recent and future directions.. Curr Opin Ophthalmol.

[17] Ferrari D, Chiozzi P, Falzoni S, Dal Susino M, Melchiorri L, Baricordi OR, Di Virgilio F (1997). Extracellular ATP triggers IL-1 beta release by activating the purinergic P2Z receptor of human macrophages.. J Immunol.

[18] Eschke D, Wüst M, Hauschildt S, Nieber K (2002). Pharmacological characterization of the P2X_7_ receptor on human macrophages using the patch-clamp technique.. Naunyn Schmiedebergs Arch Pharmacol.

[19] Frishman LJ, Shen FF, Du L, Robson JG, Harwerth RS, Smith EL, Carter-Dawson L, Crawford ML (1996). The scotopic electroretinogram of macaque after retinal ganglion cell loss from experimental glaucoma.. Invest Ophthalmol Vis Sci.

[20] Livak KJ, Schmittgen TD (2001). Analysis of relative gene expression data usingreal-time quantitative PCR and the 2^-ΔΔCT^ Method.. Methods.

[21] Brubaker RF (1996). Delayed functional loss in glaucoma. LII Edward Jackson Memorial Lecture.. Am J Ophthalmol.

[22] Hughes WF (1991). Quantitation of ischemic damage in the rat retina.. Exp Eye Res.

[23] Rosenbaum DM, Rosenbaum PS, Gupta H, Singh M, Aggarwal A, Hall DH, Roth S, Kessler JA (1998). The role of the p53 protein in the selective vulnerability of the inner retina to transient ischemia.. Invest Ophthalmol Vis Sci.

[24] Lam TT, Abler AS, Tso MOM (1999). Apoptosis and caspases after ischemia-reperfusion injury in rat retina.. Invest Ophthalmol Vis Sci.

[25] Kakurai K, Sugiyama T, Kurimoto T, Oku H, Ikeda T (2013). Involvement of P2X_7_ receptors in retinal ganglion cell death after optic nerve crush injury in rats.. Neurosci Lett.

[26] Li A, Leung CT, Peterson-Yantorno K, Stamer WD, Mitchell CH, Civan MM (2012). Mechanisms of ATP release by human trabecular meshwork cells, the enabling step in purinergic regulation of aqueous humor outflow.. J Cell Physiol.

[27] Hu H, Lu W, Zhang M, Zhang X, Argall AJ, Patel S, Lee GE, Kim YC, Jacobson KA, Laties AM, Mitchell CH (2010). Stimulation of the P2X_7_ receptor kills rat retinal ganglion cells in vivo.. Exp Eye Res.

[28] Sugiyama T, Oku H, Shibata M, Fukuhara M, Yoshida H, Ikeda T (2010). Involvement of P2X_7_ receptors in the hypoxia-induced death of rat retinal neurons.. Invest Ophthalmol Vis Sci.

[29] Zhang X, Zhang M, Laties AM, Mitchell CH (2005). Stimulation of P2X_7_ receptors elevates Ca^2+^ and kills retinal ganglion cells.. Invest Ophthalmol Vis Sci.

[30] Zhang X, Zhang M, Laties AM, Mitchell CH (2006). Balance of purines may determine life or death of retinal ganglion cells as A3 adenosine receptors prevent loss following P2X_7_ receptor stimulation.. J Neurochem.

[31] Zhang X, Li A, Ge J, Reigada D, Laties AM, Mitchell CH (2007). Acute increase of intraocular pressure releases ATP into the anterior chamber.. Exp Eye Res.

[32] Reigada D, Lu W, Zhang M, Mitchell CH (2008). Elevated pressure triggers a physiological release of ATP from the retina: Possible role for pannexin hemichannels.. Neuroscience.

[33] Berger S, Savitz SI, Nijhawan S, Singh M, David J, Rosenbaum PS, Rosenbaum DM (2008). Deleterious role of TNF-α in retinal ischemia-reperfusion injury.. Invest Ophthalmol Vis Sci.

[34] Crosson CE, Mani SK, Husain S, Alsarraf O, Menick DR (2010). Inhibition of histone deacetylase protects the retina from ischemic injury.. Invest Ophthalmol Vis Sci.

[35] Yuan L, Neufeld AH (2000). Tumor necrosis factor-α: a potentially neurodestructive cytokine produced by glia in the human glaucomatous optic nerve head.. Glia.

[36] Yan X, Tezel G, Wax MB, Edward DP (2000). Matrix metalloproteinases and tumor necrosis factor-α in glaucomatous optic nerve head.. Arch Ophthalmol.

[37] Hangai M, Yoshimura N, Yoshida M, Yabuuchi K, Honda Y (1995). Interleukin-1 gene expression in transient retinal ischemia in the rat.. Invest Ophthalmol Vis Sci.

[38] Yoneda S, Tanihara H, Kido N, Honda Y, Goto W, Hara H, Miyawaki N (2001). Interleukin-1β mediates ischemic injury in the rat retina.. Exp Eye Res.

[39] Sanchez RN, Chan CK, Garg S, Kwong JM, Wong MJ, Sadun AA, Lam TT (2003). Interleukin-6 in retinal ischemia reperfusion injury in rats.. Invest Ophthalmol Vis Sci.

[40] Chidlow G, Wood JP, Ebneter A, Casson RJ (2012). Interleukin-6 is an efficacious marker of axonal transport disruption during experimental glaucoma and stimulates neuritogenesis in cultured retinal ganglion cells.. Neurobiol Dis.

[41] Johnson EC, Doser TA, Cepurna WO, Dyck JA, Jia L, Guo Y, Lambert WS, Morrison JS (2011). Cell proliferation and interleukin-6-type cytokine signaling are implicated by gene expression responses in early optic nerve head injury in rat glaucoma.. Invest Ophthalmol Vis Sci.

[42] Niyadurupola N, Sidaway P, Ma N, Rhodes JD, Broadway DC, Sanderson J (2013). P2X_7_ receptor activation mediates retinal ganglion cell death in a human retina model of ischemic neurodegeneration.. Invest Ophthalmol Vis Sci.

[43] Notomi S, Hisatomi T, Kanemaru T, Takeda A, Ikeda Y, Enaida H, Kroemer G, Ishibashi T (2011). Critical involvement of extracellular ATP acting on P2RX_7_ purinergic receptors in photoreceptor cell death.. Am J Pathol.

[44] Yang D, Elner SG, Clark AJ, Hughes BA, Petty HR, Elner VM (2011). Activation of P2X receptors induces apoptosis in human retinal pigment epithelium.. Invest Ophthalmol Vis Sci.

[45] Petrovski G, Ayna G, Majai G, Hodrea J, Benkő S, Mádi A, Fésüs L (2011). Phagocytosis of cells dying through autophagyinduces inflammasome activation and IL-1β release in human macrophages.. Autophagy.

[46] Lee BH, Hwang DM, Palaniyar N, Grinstein S, Philpott DJ, Hu J (2012). Activation of P2X_7_ receptor by ATP plays an important role in regulating inflammatory responses during acute viral infection.. PLoS ONE.

[47] Murphy N, Cowley TR, Richardson JC, Virley D, Upton N, Walter D, Lynch MA (2012). The neuroprotective effect of a specific P2X_7_ receptor antagonist derives from its ability to inhibit assembly of the NLRP3 inflammasome in glial cells.. Brain Pathol.

[48] Kikuchi M, Tenneti L, Lipton SA (2000). Role of p38 mitogen-activated protein kinase in axotomy-induced apoptosis of rat retinal ganglion cells.. J Neurosci.

[49] Kawahara S, Hata Y, Miura M, Kita T, Sengoku A, Nakao S, Mochizuki Y, Enaida H, Kagimoto T, Goto Y, Hafezi-Moghadam A, Ishibashi T (2007). Intracellular events in retinal glial cells exposed to ICG and BBG.. Invest Ophthalmol Vis Sci.

